# Impact of morphine use in acute cardiogenic pulmonary oedema on mortality outcomes: a systematic review and meta-analysis

**DOI:** 10.1177/17539447221087587

**Published:** 2022-03-28

**Authors:** Thivanka N. Witharana, Ranu Baral, Vassilios S. Vassiliou

**Affiliations:** Norwich Medical School, University of East Anglia, Norwich NR4 7TJ, UK; Norfolk and Norwich University Hospital, Norwich, UK; Norfolk and Norwich University Hospital, Norwich, UK; Norwich Medical School, University of East Anglia, Norwich, UK; Norfolk and Norwich University Hospital, Norwich, UK

**Keywords:** hospital mortality, morphine, pulmonary oedema

## Abstract

**Background:**

Morphine is commonly used in the management of acute cardiogenic pulmonary oedema. The European Society of Cardiology (ESC) and National Institute for Health and Care Excellence (NICE) do not recommend the routine use of opioids in acute heart failure (AHF) due to dose-dependent side effects. However, the effect of morphine remains unclear. Our study aims to investigate the link between morphine use in acute cardiogenic pulmonary oedema and mortality.

**Methods:**

PubMed and Embase databases were searched from inception to October 2021. All studies were included (randomized, non-randomized, observational, prospective and retrospective). The references for all the articles were reviewed for potential articles of interest with no language restrictions. Studies looking at in-hospital mortality along with other outcomes were chosen. The Newcastle–Ottawa scale was used to appraise the studies. Heterogeneity was assessed using *I*^2^. Meta-analysis was conducted using the Review Manager Software version 5.3 (The Nordic Cochrane Centre, The Cochrane Collaboration, 2014), by computing odds ratios (ORs) for pooled in-hospital mortality and clinical outcomes.

**Results:**

Six observational studies out of the 73 publications identified were eligible for the meta-analysis giving a total sample size of 152,859 (mean age 75, males 48%). Of these, four were retrospective analyses. The use of morphine in acute cardiogenic pulmonary oedema was associated with an increased rate of in-hospital mortality [OR = 2.39, confidence interval (CI) = 1.13 to 5.08, *p* = 0.02], increased need for invasive ventilation (OR = 6.14, CI = 5.84 to 6.46, *p* < 0.00001), increased need for non-invasive ventilation (OR = 1.85, CI = 1.45 to 2.36, *p* < 0.00001) and increased need for vasopressors/inotropes (OR = 2.93, CI = 2.20 to 3.89, *p* < 0.00001).

**Conclusion:**

Based on the observational studies, morphine use in acute cardiogenic pulmonary oedema is associated with worse outcomes. Further randomized controlled trials are needed to confirm any causative effect of morphine on mortality rates in acute cardiogenic pulmonary oedema.

## Introduction

Morphine is one of the commonly used drugs in the management of acute cardiogenic pulmonary oedema.^1^ It is recommended as a level IIb intervention under the European Society of Cardiology (ESC) guidelines to relieve dyspnoea and anxiety in the early stages of acute heart failure (AHF).^1^ Morphine helps in pulmonary oedema by reducing the preload and therefore reducing the pulmonary capillary pressure. It also reduces the afterload to a lesser extent.^2^ At a cellular level, morphine and its metabolite morphine-6-glucuronide act as agonists on the mu and kappa opioid receptors.^3^ The cation on mu receptors is thought to be associated with the side effects such as modification of the respiratory system and addiction.^3^ Both ESC and National Institute for Health and Care Excellence (NICE) recommend not to use opioids routinely in AHF due to dose-dependent side effects such as nausea, bradycardia, hypotension and respiratory depression.^1,4^ However, prognostic benefits of morphine remain unclear; whether it simply relieves acute symptoms or if it might even worsen outcomes. There is conflicting evidence regarding potentially elevated mortality risk in AHF patients receiving morphine.^5,6^ Therefore, this systematic review was conducted to find out whether there is a link between morphine use in acute cardiogenic pulmonary oedema and adverse patient outcomes and to provide up-to-date evidence, identified in a systematic approach building on existing meta-analyses.^7–9^

## Methods

The systematic review and meta-analysis was conducted and reported according to the Preferred Reporting Items for Systematic Reviews and Meta-Analysis (PRISMA) guidelines (Supplementary material, Table 2).^10^

### Search strategy

An extensive search was carried out on PubMed and Embase databases from inception to October 2021 using key search terms such as ‘pulmonary oedema’ OR ‘pulmonary edema’ OR ‘Acute heart failure’ AND ‘Morphine’ AND ‘Mortality’. Mortality was our primary outcome measure. A snowballing method was used to the references of trials to broaden the search. No language or study design restrictions were applied.

### Eligibility criteria

All studies (e.g. randomized, non-randomized, observational, prospective and retrospective) that reported the effects of morphine use in acute cardiogenic pulmonary oedema in adults (age  > 18) were included. Conference abstracts were excluded as there was inadequate detail for quality assessment. The primary outcome was in-hospital mortality.

### Data analysis

All studies identified in the search were screened by two authors (T.N.W. and R.B.) individually using titles and the abstracts. Disagreements were adjudicated by a third author (V.S.V.). Any trial with the potential of fulfilling our inclusion criteria underwent full-text evaluation. From each trial included in the systematic review, following data were extracted: study design, sample size, average age, percentage of males, presence of comorbidities such as ischaemic heart disease (IHD), hypertension, diabetes mellitus, chronic lung disorders, atrial fibrillation (AF), serum sodium levels, serum haemoglobin (Hb) levels, serum brain natriuretic peptide levels, ejection fraction, number of participants who received morphine and number of participants in the control group.

Meta-analysis was conducted using the Review Manager Software version 5.3 (The Nordic Cochrane Centre, The Cochrane Collaboration, 2014), by computing odds ratios (ORs) for pooled in-hospital mortality and clinical outcomes. We prospectively decided to use a random effect model. Heterogeneity was assessed using the *I*^2^. In terms of the quality of the studies, we hypothesized that according to the methodological quality of the studies, the effect size may vary. To find out if any one study carried significant weight, we conducted the analysis by excluding one study at a time. Newcastle–Ottawa Scale was used to appraise the studies.

## Results

**Figure 1.**
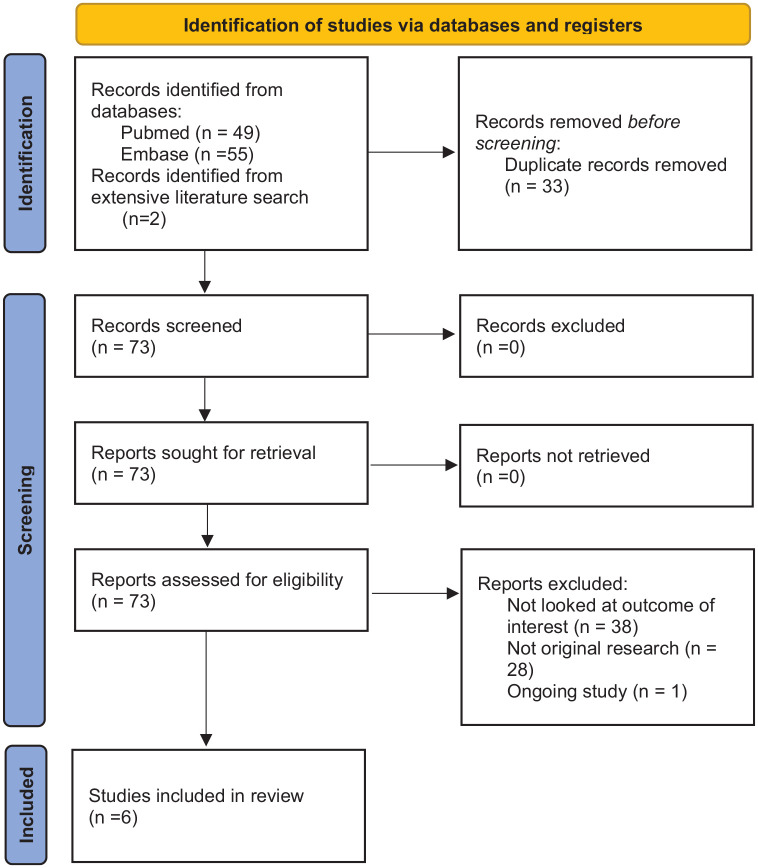
Preferred Reporting Items for Systematic Reviews and Meta-Analyses (PRISMA) flow diagram of the trial selection process.

A total of 106 publications were identified from database search (Figure 1).^11^ After de-duplication of 33 studies, 73 studies underwent screening. Some 67 further studies were excluded. In total, six studies were used in the meta-analysis.

This systematic review meta-analysis consists of a total sample size of 152,859 participants with a mean age of 75 years, males 48%, IHD 2%, diabetes 44%, chronic lung conditions 31% and AF 31%. The characteristics of the six studies are shown in Table 1.

**Table 1.** Characteristics of the selected studies.

Study name	Study design	Sample size *n*	Male %	Mean age years	Outcomes studied
Caspi O *et al*.^12^	Retrospective Observational cohort	1344	41	78	Invasive ventilation In-hospital mortality Noninvasive ventilation Inotrope use Acute kidney injury
Miró Ò *et al*.^13^	Prospective Observational Cohort	550	57	81	In-hospital mortality 30-day mortality Need for inotropes/vasopressors Need for noninvasive ventilation Need for mechanical ventilation
Dominguez-Rodriguez A *et al*.^14^	Retrospective Observational Cohort	991	28	67	In-hospital mortality
Iakobishvili *et al*.^5^	Prospective Observational Cohort	2336	45	76	In-hospital mortality Need for IV inotropes
Peacock W *et al*.^6^	Retrospective Observational cohort	147,362	48	75	In-hospital mortality Hospitalization length ICU admission ICU length of stay Mechanical ventilation
Fiutowski *et al*.^15^	Retrospective Observational Cohort	276	46	70	In-hospital mortality

ICU, intensive care unit; IV, intravenous.

Patient demographics including presence of comorbidities (Tables 2 and 3) were similar in the patient samples who received morphine (intervention) compared with those who did not receive morphine (control) across all studies. For the Dominguez-Rodriguez *et al*.^16^ and Fiutowski *et al*.^15^ studies, the demographics of intervention and control groups were not reported.

**Table 2.** Patient demographics; morphine group *versus* non-morphine group.

Demographics	Morphine group (intervention) *n* = 21,947 (%)	Non-morphine group (control) *n* = 129,645 (%)
Age	73	75
Male	10,286 (47)	62,769 (48)
IHD	8414 (38)	44611 (34)
HTN	16,540 (75)	95,024 (73)
DM	9957 (45)	57,272 (44)
CLD	7163 (33)	39,797 (31)
AF	6171 (28)	40,885 (32)
Sodium	138	139
Hb	12	12

AF, atrial fibrillation; CLD, chronic lung disease; DM, diabetes mellitus; Hb, haemoglobin; HTN, hypertension; IHD, ischaemic heart disease.

**Table 3.** Patient demographics.

Study name	Year	Sample size	Mean age (years)	Male (%)	IHD	HTN	DM	CLD	AF
Caspi O	2019	1344	78	41	366	1013	730	189	557
Miró Ò	2017	550	81	57	202	481	266	118	231
Dominguez-Rodriguez A	2016	991	67	28	221	620	425	161	301
Iakobishvili Z	2011	2336	76	45	922	1785	1207	451	680
Peacock W	2007	147,362	75	48	1,586	108,285	65,026	46,202	45,588
Fiutowski M	2004	276	70	46	262	191	97	–	37

AF, atrial fibrillation; CLD, chronic lung disease; DM, diabetes mellitus; Hb, haemoglobin; HTN, hypertension; IHD, ischaemic heart disease.

**Figure 2.**
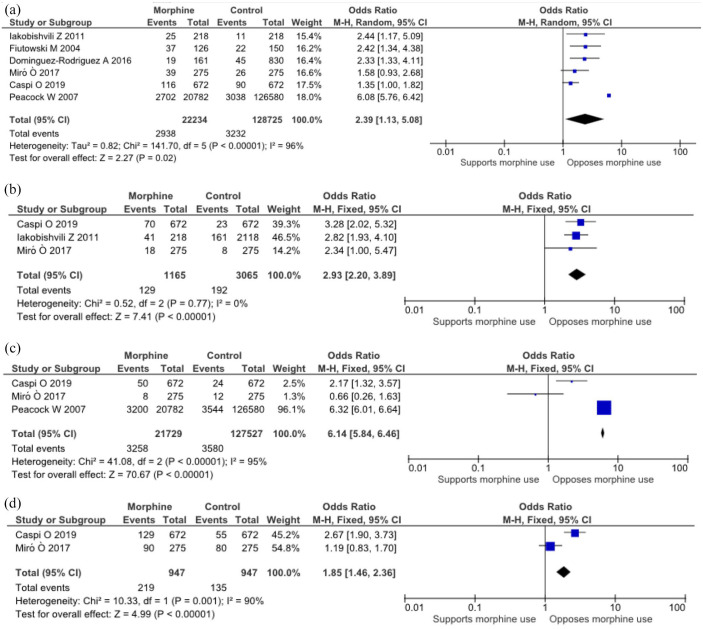
(a) Morphine use and in-hospital mortality. Morphine use in acute cardiogenic pulmonary oedema is associated with a 2.39 times increase in in-hospital mortality [odds ratio (OR) = 2.39, 95% confidence interval (CI) = 1.13 to 5.08]. (b) Morphine use and need for inotropes/vasopressors. Morphine use in acute cardiogenic pulmonary oedema is associated with 2.93 times increased need for inotropes/vasopressors (OR = 2.93, 95% CI = 2.20 to 3.89). (c) Morphine use and need for invasive ventilation. Morphine use is associated with a 6.14-fold increase in the need for invasive ventilation (OR = 6.14, 95% CI = 5.84 to 6.46). (d) Morphine use and need for noninvasive ventilation. Morphine use is associated with a 1.85-fold increase in the need for non-invasive ventilation (OR = 1.85, 95% CI = 1.46 to 2.36).

All six studies examined the relationship between morphine use in acute cardiogenic pulmonary oedema and in-hospital mortality. Our meta-analysis showed that morphine use in acute cardiogenic pulmonary oedema is associated with a significant 2.39 times increase in in-hospital mortality [OR = 2.39, 95% confidence interval (CI) = 1.13 to 5.08, *p* = 0.02; Figure 2(a)]. This was also true for the subgroup analysis performed on the two studies that used propensity score–matched analysis with OR = 1.40, 95% CI = 1.08–1.82, *p* = 0.01 (as shown in Figure 1 in supplementary material).

Furthermore, pooled analysis of three studies^5,12,13^ that examined the relationship between morphine use and the need for inotropes/vasopressors in acute cardiogenic pulmonary oedema showed almost a threefold significant increase in need for inotropes/vasopressors in the morphine group as compared with the control [Pooled OR = 2.93, 95% CI = 2.20 to 3.89, *p* < 0.00001; Figure 2(b)].

The association between morphine use and need for invasive ventilation was examined in three studies. Two out of the three studies showed an increase in the need for invasive ventilation in the morphine group while one showed an increase in need in the control group (Figure 2(c)). Our meta-analysis was carried out to reveal that overall, morphine use is associated with a 6.14-fold increase in the need for invasive ventilation compared with non-morphine use (OR = 6.14, 95% CI = 5.84 to 6.46, *p* < 0.00001). Besides, two studies reported data for noninvasive ventilation. Pooled analysis demonstrated an overall increase of 1.85 times in the patients on morphine compared with controls [OR = 1.85, 95% CI = 1.46 to 2.36, *p* < 0.00001; Figure 2(d)].

**Figure 3.**
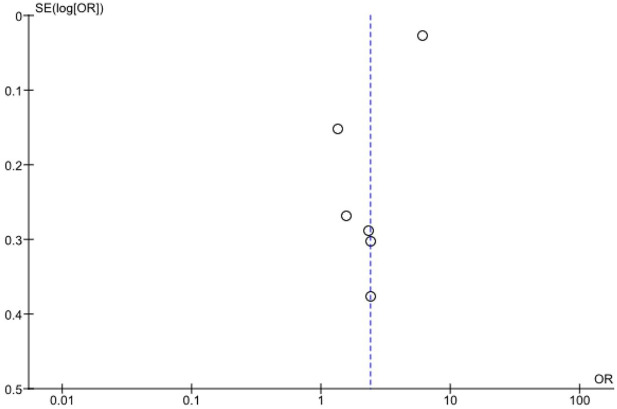
Funnel plot.

**Figure 4.**
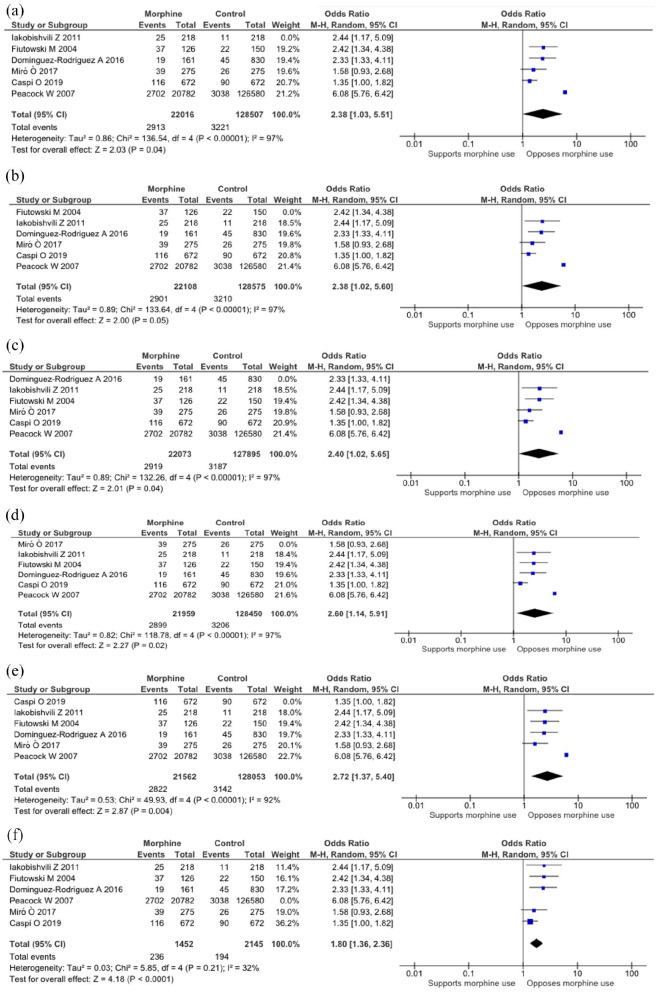
Sensitivity analysis for in-hospital mortality. (a) Sensitivity analysis with Iakobishvili study excluded. (b) Sensitivity analysis with Fiutowski study excluded. (c) Sensitivity analysis with Dominguez-Rodriguez study excluded. (d) Sensitivity analysis with Miró study excluded. (e) Sensitivity analysis with Caspi study excluded. (f) Sensitivity analysis with Peacock study excluded.

Funnel plot and sensitivity analyses were undertaken and shown in Figures 3 and 4, respectively. The appraisal standards assessed by Newcastle–Ottawa Scale are shown Table 1 in supplementary material.

## Discussion

Our systematic review and meta-analysis demonstrated increased in-hospital mortality with morphine use in acute cardiogenic pulmonary oedema. Morphine use is also seen to be associated with an increased need for inotropes/vasopressors, invasive ventilation and noninvasive ventilation. Overall, it is linked to significantly worsening outcomes in patients with acute cardiogenic pulmonary oedema.

None of the included studies were randomized controlled trials (RCTs). So, we are unable to confirm whether the groups were similar or not. However, it is likely that the groups of patients who received morphine were more unwell with limitation of therapeutic effort. This could well have been a confounder as the studies were observational. Lack of RCTs can be explained by the fact that morphine use is usually associated with use in severely unwell patients and therefore randomized or placebo-controlled trials around its usage is rarely approved. However, propensity score–matched analysis provides insight into these scenarios where higher levels of evidence are lacking. In our meta-analysis, two of the primary researches are propensity score–matched analyses. The meta-analyses of these studies on in-hospital mortality also showed adverse outcomes with morphine use (OR = 1.40, 95% CI = 1.08–1.82, *p* = 0.01) as shown in Figure 1 in supplementary material.

Currently, evidence supporting the use of morphine in this patient group is not available. Hence, current practise uses a therapeutic approach where a potentially harmful class of drugs is used in these acutely ill patients.^17^ The ESC suggests cautious use of morphine in patients with severe dyspnoea, mainly in those with acute pulmonary oedema. Similarly, the American Heart Association/American College of Cardiology recommends the use of morphine therapy only in palliative care of end-stage heart failure.^18^ Evidence for the use of morphine in acute cardiogenic pulmonary oedema in the form of large RCTs is lacking.^17^

The use of morphine in dyspnoea and anxiety is well known.^14^ In acute cardiogenic pulmonary oedema, there is increased vascular resistance due to release of endogenous catecholamines.^17^ Morphine with its vasodilatory properties results in decreased venous tone which reduces vascular return to the right heart and eventually a reduced right ventricular output.^12^ This allows the weaker left ventricle to function at a lower filling pressure. This will also cause hypotension and a decrease in cardiac output. The decrease in cardiac output is perhaps related to an increased need for intensive care unit (ICU) admissions and endotracheal intubations.^11^

Our meta-analysis revealed an increased need for both invasive and noninvasive ventilation. The beneficial effect of morphine use in acute cardiogenic pulmonary oedema seems to be the anxiolytic effect and the systemic vascular resistance. However, it may be possible that alternative therapy, such as benzodiazepines for anxiety, provides similar effects without the increased adverse effects seen in morphine.^11^ Further research will of course be needed to test the efficacy and safety of these therapies in AHF.

The detrimental effects of morphine can also be partially explained by its interactions with other medications. Morphine when combined with antiplatelets such as ticagrelor, clopidogrel and prasugrel demonstrated a delayed activity. Besides, there is evidence of a decreased heart rate and consequently cardiac output with morphine.^19^ This can potentially decrease myocardial perfusion and lead to ischaemia and cardiogenic shock. These cardiac effects may be fatal in patients with IHD who are already at risk of heart failure.^19^

The effects of morphine remain controversial. Midazolam *versus* Morphine in Acute Pulmonary Oedema (MIMO) trial, a multicentre prospective randomized study that aims to assess the safety of morphine in acute cardiogenic pulmonary oedema, will address the gaps in our knowledge in the field.^20^ It is important to note that this RCT does not have a control group, as it is unethical not to provide symptomatic relief in these patients.

## Study limitations

Five out of the six studies used in our meta-analysis are retrospective studies. Only observational studies are available. Due to the lack of evidence from RCTs, it is difficult to prove causality. Nevertheless, this study shows a significant association between morphine use and mortality. Therefore, it essentially allows us to risk-stratify the patients who receive morphine (at the discretion of the clinical team) and identify these patients as ‘high risk’ and therefore provide increased vigilance and therapy. Furthermore, the total dose of morphine used and the timings of administration in the patients were not given in the studies making it impossible to find out if outcomes were affected by dose differences. As such, we used a binary measure of any morphine or no morphine used. In addition, it was not possible to identify whether the causes of in-hospital mortality in the participants were of a cardiac origin or not. Nevertheless, evidence suggests that most patients admitted with acute pulmonary oedema die from heart failure–related causes,^21^ so a cardiac-related death is more likely.

## Conclusion

In-hospital mortality along with the use of inotropes and invasive and noninvasive ventilation were higher in patients with acute cardiogenic pulmonary oedema who received morphine compared with those who did not receive morphine. However, due to lack of evidence from RCTs, a causative effect could not be investigated. Hence, until randomized data are available, our study supports the current guidelines in suggesting cautious use of morphine in the management of acute cardiogenic pulmonary oedema.

## Supplemental Material

sj-docx-1-tak-10.1177_17539447221087587 – Supplemental material for Impact of morphine use in acute cardiogenic pulmonary oedema on mortality outcomes: a systematic review and meta-analysis Click here for additional data file. Supplemental material, sj-docx-1-tak-10.1177_17539447221087587 for Impact of morphine use in acute cardiogenic pulmonary oedema on mortality outcomes: a systematic review and meta-analysis by Thivanka N. Witharana, Ranu Baral and Vassilios S. Vassiliou in Therapeutic Advances in Cardiovascular Disease
